# Enhancing workplace competence acquisition through a persuasive system

**DOI:** 10.1007/s10257-022-00571-6

**Published:** 2022-09-28

**Authors:** Stefano Za, Eusebio Scornavacca, Jessie Pallud

**Affiliations:** 1 Department of Management and Business Administration, University “G. d’Annunzio” of Chieti-Pescara, Viale Pindaro 42, 65127 Pescara, Italy; 2 Arizona State University, 1120 South Cady Mall, Tempe, AZ 85287-5603 USA; 3 EM Strasbourg Business School, Humanis (UR7308), 61 Avenue de la Forêt Noire, 67085 Strasbourg Cedex, France

**Keywords:** Competence acquisition, Persuasive system, Mobile app, Design Science Research, Employee development

## Abstract

The continuous development of individual competences is a fundamental instrument for organizations aiming to achieve long-term competitive advantage. Digital technologies can play a pivotal role in competence acquisition by facilitating the learning process. This paper describes the design, development, and evaluation process of a competence acquisition mobile app incorporating persuasive systems principles. A pilot study involving managers from an IT consulting firm was used to evaluate the artifact. This was followed by a full-scale deployment of the mobile app with management and staff of a supermarket chain. The results indicate that the use of the mobile app effectively improved users’ learning process outcomes, as well as their ability to deploy the new competence. Theoretical and practical contributions are discussed.

## Introduction

Organizations have been increasingly investing in competence development programs for their employees in order to ensure they remain competitive (Camps et al. 2016; North-Samardzic et al. 2014). The corporate training market (including technical and non-technical courses for employees) is expected to grow by $ 14.06 billion during 2021–2025, progressing at a compound annual growth rate (CAGR) of over 8% during that period (Reportlinker 2021). The growing business spending in competence acquisition and competence development demonstrates that companies recognize the strategic importance of investing in this area. Employee development, a mission generally devoted to the HR function, is a strategic issue that can impact on the extent to which an organization has the capabilities to remain competitive.

Especially during the global pandemic, digital technologies have been widely deployed for training and developing human resources.^1^ According to a recent report, most organizations will continue to shift budgets away from instructor-led training (ILT) to online learning: 73% of learning and development professionals expect to spend less on ILT and 79% expect to spend more on online learning (Linkedin Learning 2021). As a result, the development of effective digital tools to support competence acquisition have become a crucial issue in the realm of HR management (Ley 2020).

Nonetheless, there are many shortcomings related to e-learning systems, such as technological limitations (e.g., bandwidth), personal issues (e.g., lack of IT skills), limitations compared to face-to-face learning (e.g., lack of physical interaction) and design limitations (e.g., relevance of the online content) (Wong 2007).

Therefore, there is still a need for researchers and professionals to pursue innovative approaches in terms of design and delivery of human resource development. Furthermore, how to design and develop information systems for employee training in the workplace still represents an important research gap that deserves further attention (McPherson and Nunes 2006; Wong 2007; Za and Braccini 2012; Zhang et al. 2004). The emergence of “persuasive technologies”—interactive computing systems designed to influence people's attitudes and behaviors towards a specific target—also represent a unique opportunity for improving employee competence acquisition processes as they have the capacity to foster user interest and engagement over time (Fogg 2003). Persuasive systems could be defined as “computerized software or information systems designed to reinforce, alter, or shape attitudes and/or behaviors without resorting to coercion or deception” (Cyr et al. 2018).

Hamari et al. (2014) found that the number of papers on persuasive systems has increased since 2005. Those technologies exert power on individuals because they incorporate social cues such as physical and psychological cues, language, social dynamics, and social roles. Prior research has found that persuasive systems could be used in the learning context to change learners’ behaviors, but it has not provided any technological artifact to assess such change (Filippou et al. 2016; Widyasari et al. 2019).

While the choice of medium and design of the digital artifact are important elements in the pursue of human resource development, the actual selection of the competences to be developed is a fundamental step in this process. Among the top 10 skills that learning and development experts deemed important in 2021, 9 out of 10 were soft skills (Linkedin Learning 2021): resilience and adaptability come first, then digital fluency, communication, and emotional intelligence.

Elements such as volatility, uncertainty, complexity and ambiguity (VUCA) of business environments require from workers to be more adaptable and resilient as well to develop more robust soft skills (Lepeley 2021). With the Covid 19 crisis, for example, Lepeley (2021) argues that soft skills have become the focus of attention of many companies: “hard times are corroborating the need for soft skills” (p. 6). Indeed, not only do soft skills have positive effects on human management, performance and competitiveness, but they also contribute to workers’ well-being (Lepeley 2021). This indicates that the bulk of employee development programs should be focused on soft skills. Research has also demonstrated that employers (Ritter et al. 2018) and higher education institutions (Succi and Canovi 2020) have been increasingly prioritizing soft skills. According to Kyrousi et al. (2022), soft skills are “transferable across careers in a range of business functions and are non-technical social skills”. They include a number of skills such as “listening, communication, teamwork, time management, self-management, empathy, integrity, flexibility, emotional intelligence” (Anthony and Garner 2016, p. 361). Recently, Kyrousi et al. (2022) found that communication is among the top three soft skills for employability and, according to Lepeley (2021), effective social communications is at the heart of high performing organizations. Our study focuses on the skill of “providing feedback to colleagues”, the strategic importance of good communication in the workplace.

The process of developing a persuasive system to facilitate soft skills acquisition in the workplace can be substantiated through a Design Science Research (DSR) approach. DSR provides a robust process for the creation of successful IS artifacts (Gregor and Hevner 2011; Peffers et al. 2008; Riemer and Seidel 2013; vom Brocke et al. 2020). It seeks to enhance technological and scientific knowledge though the creation of innovative artifacts that solve problems and improve the environment in which they are instantiated (vom Brocke et al. 2020).

As a result, this paper aims to explore how persuasive technologies can facilitate soft skills acquisition in the workplace. In order to achieve this goal, we adopted a Design Science Research (DSR) approach based on Peffers et al. (2008) to design, develop and evaluate a competence acquisition mobile app.

The contributions of this research should be of interest to IS as well as Human Resources (HR) specialists who examine learning strategies in the workplace with technologies. This research makes contributions to the IS discipline by providing an integrated DSR framework that combines several models. Furthermore, it develops both an *abstract* artefact that is twofold: (i) a specific training method for fostering competences acquisition and (ii) the process for creating the exercises used for the training; and a *material* artifact, specifically the mobile app for competence acquisition. This study makes contribution to practice by applying the developed mobile app into a workplace setting and demonstrating the potential of persuasive technologies to enhance soft skills acquisition.

The structure of the paper is as follows. First, the paper reviews the literature on employee development, and it identifies the limitations of traditional methods used for competence acquisition. Second, it introduces two different perspectives on persuasive systems and atomic competence that could overcome the limitations aforementioned. Third, it describes an integrated Design Research framework for designing an artifact fostering competence acquisition. Fourth, the development and evaluation of the competence acquisition mobile app is detailed. Finally, theoretical and managerial contributions are discussed, as well as limitations and opportunities for future research.

## Theoretical background

The first part of this section aims to provide an overview on competence acquisition, focusing specifically on some relevant issues to be taken into consideration concerning employee development at the workplace. The second part introduces the concept of persuasive system and the relative characteristics integrated in the design process of the competence acquisition mobile app.

### Competence development

The concept of competence was first introduced in the human resources field by David McClelland (1973) who argued that competences should be assessed instead of intelligence to better account for job success. He also provided six principles to test competences more accurately.

Boyatzis (2008, p. 6) defines a competence as “a capability or ability”. But there is no convergent definition of the term “competence” in the human resources management literature (Le Deist and Winterton 2005). For example, Draganidis and Mentzas (2006) list a dozen of definitions of the term in the academic and practitioner literature in order to provide their own definition of competency: "a combination of tacit and explicit knowledge, behaviour and skills, that gives someone the potential for effectiveness in task performance” (p. 53). Actually, competence is often referred to as a combination of different factors such as knowledge, skills, and/or individual characteristics. Le Deist and Winterton (2005) propose a holistic competence model composed of three fundamental dimensions: cognitive (knowledge and understanding), functional (skills) and social (behaviour and attitude). While the distinction of the three fundamental dimensions could be analytically identified, in practice, the competence of an individual is the result of the combination of these three fundamental dimensions. Those three dimensions are also identified by Boyatzis and Ratti (2009, p. 824): “Competencies are a behavioural approach to emotional, social, and cognitive intelligence”. In addition, competencies may be fostered in an organization through training and development (Boyatzis 2008; McClelland 1998), aiming to improve the performance of specific tasks. The extent to which the training is effective may be measured on the basis of pre-established criteria (Boyatzis 1982). Research indicates that competencies can be used to distinguish outstanding top executives and managers, as both positions tend to exhibit distinct social, emotional, and cognitive intelligence competences (Boyatzis and Ratti 2009).

Moreover, in this paper we focus specifically on the concept of atomic competence (Baldoni et al. 2011). Atomic competences (also known as micro competences) can be understood as the smallest element of a competence (Pedraza-Jimenez et al. 2004; Valverde-Albacete et al. 2003). Once a competence is identified, it should be verified if it corresponds to an atomic competence (Baldoni et al. 2011) or if it is composed by a set of atomic competences. In the second case, the competence is further broken up until a single atomic competence is reached. For each micro competence, it is necessary to define its fundamentals characteristics that can be translated into simple tasks.

We adopt Howell's (1982) five-stage competence model as it provides a quite comprehensive framework that covers a wide variety of competences valuable in an organizational setting—from behaviours related to IS security (Thomson et al. 2006) to skills required for cross-cultural team working (Barnes et al. 2000; Tung 1993). The key assumption underlying the model is that people will respond to training when they are aware of its value and need (Thomson et al. 2006). The five stages of competences are described below:

1. *Unconscious Incompetence*: At this stage employees are unaware of the existence of a specific competence. Unintentionally, they may possess an undesired behaviour regarding a specific task. They do not necessarily perceive a deficiency in their skills set. As a result, there is a need to become conscious about competence before starting the specific learning process.
2. *Conscious Incompetence*: employees are aware about the missing competence required to carry out a desirable behaviour or to perform a specific task correctly. Often this state of awareness occurs when the individual lacks parity with other colleagues’ competence set.
3. *Conscious Competence*: employees need to make a mental effort (think through) in order to be able to perform a task competently. The desired behaviour is still not spontaneous or “innate”. This phase is characterized as being "thoughtful-analytical", where each problem is considered and analysed at a time.
4. *Unconscious Competence*: the task is performed correctly and spontaneously. The correct behaviour is part of the employees’ subconscious. In this case, employees could have some difficulty to really explain how the task needs to be done since it has become mostly ingrained in their competence set.
5. *Unconscious Super Competence*: in this stage employees have practiced and internalized effective ways to accomplish tasks. They become extremely (or super) competent in accomplishing these tasks.

In order to foster competences development, companies tend to rely increasingly on e-learning systems (Capece and Campisi 2013; Cheng et al. 2014; Costello and McNaughton 2016; Mohammadyari and Singh 2015). The rapid pace of mainstream adoption of new forms of ubiquitous digital devices such as smartphones and tablets are enabling an ubiquitous learning environment that can be paramount for the development of competence acquisition tools (Chu and Lo 2011; Kaganer et al. 2013; Lyytinen et al. 2004). As users are able to accomplish a multitude of tasks and interact fluidly in an ubiquitous ecosystem (Carillo et al. 2014), they become empowered of their own individual learning process. For instance, an interesting example of innovative design and delivery of competence development is Google’s Primer mobile app. It aims at training employees in a fast and easy way with a 5-min module. It allows people to get free training on marketing basics anywhere and at their own pace.

Capece and Campisi (2013) demonstrated that e-learning training programs can generate positive attitudes towards technology-mediated education. They conducted a case study in a multinational company where they collected 5083 questionnaires. Respondents had to train themselves on the Sarbanes–Oxley Act through a handful of e-learning modules and, overall, the results indicate that perceived usefulness and ease of use of the modules lead to employee satisfaction. More recently, Dachner et al. (2021) identified 5 firm- and job-related trends that require rethinking employee development in the workplace. First, employees have to deal with more complex and dynamic tasks that necessitate soft skills (such as creativity) and problem-solving strategies. They also often face time constraints because of the imperative of new competency requirements. Second, employees must deal with increasing work demands, very often longer hours of work, more responsibilities and travelling. Third, they have less opportunities for upward mobility. Fourth, technologies have become pervasive in the workplace and central in employees’ communications and interactions. Five, since employees change jobs more regularly, they have to continuously learn new knowledge and skills.

These five trends allow us to conclude that employee development should be more focused on the acquisition of soft skills and that it needs to take into account time and space constraints that employees face. Dachner et al. (2021) also recommend proactivity in competence development programs so that employees feel in personal control of what they learn. Proactive learning should also help employees “feel capable, sufficiently motivated, and energized” (ibid, p. 7).

Prior research has mainly focused on “learning strategies, barriers, facilitators and outcomes” (Crouse et al. 2011), thus providing very little attention to design issues. This lack of understanding on how to best design and develop mobile systems for employee training in the workplace represents an important research gap which this study aims to contribute to (McPherson and Nunes 2006; Wong 2007; Zhang et al. 2004). In the next section, we suggest a renewed approach, based on persuasive systems, to develop e-learning modules for workplace training that acknowledge the five trends identified by Dachner et al. (2021). The objective is to develop a system for supporting the acquisition (learning process) of a specific competence—exploiting the ubiquity nature of digital technologies. As such, a mobile app could represent an adequate solution (Pereira and Rodrigues 2013; Pimmer and Pachler 2014).

### Persuasive systems

Persuasive systems have increasingly become the object of industry and scholarly research (Byrnes 2015). Persuasive systems respond to a critical issue of making users go beyond simply adopting technologies (Venkatesh and Davis 1996) to actually engage with them over an extended span of time in order to develop desirable behaviours and organizational practices (Peng et al. 2019).

The underlying mechanism of persuasive information technologies is persuasion. It is known to be an effective method to imply a voluntary change of behaviour in individuals (Berdichevsky and Neunschwander 1999). It differs from coercion or deception since persuasion does not force a change of behaviour nor stipulate the use of misinformation. Media and digital technologies (from billboards to television and the Internet) play a relevant role in “facilitating the delivery of persuasive messages to purchase, donate, vote, concede, or act” (IJsselsteijn et al. 2006, p. 1).

**Fig. 1:**
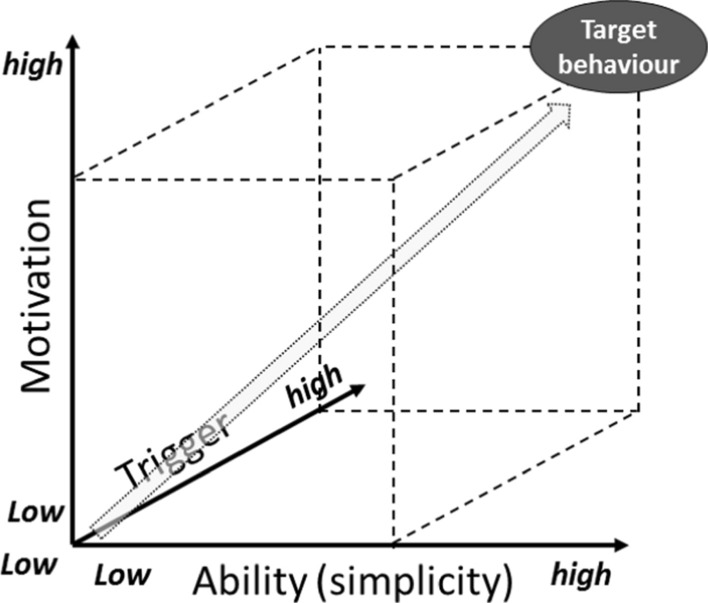
The three main factors for persuading people to perform a specific behaviour (Adapted from Fogg, 2009a, b)

Usually, the process of persuading individuals to perform a target behaviour requires the simultaneous presence of three factors: motivation, ability (or simplicity), and triggers (Fogg 2009a). Figure 1 presents the adapted Fogg’s conceptual framework illustrating the relationships among these three factors.

High motivation (the reason to carry out the target behaviour) and high ability (often related to “simplicity” to accomplish a specific task) are necessary to reach the target behaviour but are not enough. Trigger is the third fundamental factor that is necessary as well and at the same time: indeed, “without an appropriate trigger, behaviour will not occur even if motivation and ability are high” (Fogg 2009a). A trigger can have different forms, such as an alarm or a notification on a digital technology. The main objective is to draw the individual’s attention to perform a specific task.

The emergence of “persuasive technologies”—interactive computing systems designed to influence people's attitudes and behaviours towards a specific target—also represent a unique opportunity for improving the process of employee competence acquisition as they have the capacity to foster user interest over time (Fogg 2003).

Those technologies exert power on individuals because they incorporate social cues such as physical and psychological cues, language, social dynamics, and social roles. For instance, Fogg’s research has shown that simulators, avatars or video game consoles can be considered as persuasive systems for their physical cues: the attractive interfaces of these devices especially contribute to increase their persuasive power. Indeed, “a computing technology that is visually attractive to target users is likely to be more persuasive as well.” (Fogg 2003, p. 93). Smartphones also qualify as persuasive technologies because they possess all of the social cues identified by Fogg (2003). In addition, the ubiquity and multiple functionalities of the devices contributes to social presence at a large scale (Cui et al. 2021).

The Elaboration Likelihood Model—ELM—(Petty et al. 1983) is the most common framework for developing persuasion and it is applicable to various sources and context variables (Kitchen et al. 2014). According to the ELM, persuasion may be induced through a central route or a peripheral route: the former represents the *content* and it is related to the interest stimulated by the content of the message received by the users, the latter represents the *format* and it is related to cues such as attractiveness of the message source (Petty and Cacioppo 1986; SanJosé-Cabezudo et al. 2009). The state of “elaboration likelihood” (EL) is the extent to which individuals choose to process the information provided by a source. If the EL is high, individuals are likely to process the message received, since they are interested in the arguments of the message (central route). On the other hand, if the EL is low users need to be motivated by using extra stimuli (peripheral route) (Cyr et al. 2018). The state of EL determines the route through which persuasion may occur (Petty et al. 1983; Petty and Cacioppo 1986). Furthermore, the combination of both routes can “act jointly and significantly to impact attitudes and intentions in individuals’ behavior” (SanJosé-Cabezudo et al. 2009, p. 306), where one route—usually the peripheral—might enhance the effects of the other one—often the central (Cyr et al. 2018; SanJosé-Cabezudo et al. 2009).

One way to foster user engagement is to incorporate persuasive elements in the design of a digital solution aimed at fostering behavioral competency acquisition (Oinas-Kukkonen and Harjumaa 2009). Specifically, the artefact should incorporate persuasive elements for further stimulating the user engagement in the digital learning process (Chatterjee et al. 2018; Filippou et al. 2016) and for influencing human behaviour in a positive way, motivating users to engage in self-management and triggering behavioural changes (Asbjørnsen et al. 2019). For instance, Filippou et al. (2016) created two statistical models that help understanding how students and alumni manage their academic performance. Those models are presented as the basis for the development of a persuasive system to improve students’ learning. While the authors did not develop a persuasive system per se, they rely on Persuasive Systems Design to influence students’ behaviour in order to improve their academic performance. Along similar lines, Widyasari et al. (2019) suggests the application of Web 2.0-based e-learning system with persuasive features can be an effective mean to positively change learners’ behaviour. More precisely, they conducted a survey in a class of 30 students to uncover if persuasive features could change users’ acceptance of new learning methods. Their findings showed mixed feelings regarding user involvement and intention to use online learning. However, both studies did not provide any technological artifact or precise features to impact learning, nor do they provide measures of engagement or effectiveness, gaps we try to fill in with that study. According to Fogg (2009a, b), persuasive systems should target a single behaviour to avoid flooding users with excessive information and to ensure its effectiveness. Therefore, selecting one atomic competence for our DSR approach aligns with Fogg’s recommendation and ensures the persuasiveness of the training program.

## The design science research process

The main goal of Design Science Research is the creation of successful IS artifacts (Gregor and Hevner 2011; Hevner et al. 2004; Peffers et al. 2008; Riemer and Seidel 2013; vom Brocke et al. 2020). Those artifacts can be virtual (e.g. databases, programs) or physical products (e.g., mobile device) or methods such as a prototyping methodology or an IS management strategy. As the word “design” is both a noun and a verb, a theory can be about both the principles underlying the form of the design and also about the act of implementing the design in the real world (Gregor and Jones 2007). Gregor and Jones (2007) also underline the relationships between the two main types of IS/IT artifacts, namely: (i) the *materials* artifacts, such as an instantiated product or an instantiated method: they have a physical existence in the real world; and (ii) the *abstract* artifacts, such as methods, constructs, and models: they usually need to be communicated in words or diagrams for having a physical existence. Individuals conceptualize and describe *abstract* artifacts in general terms, providing theories used for supporting the building of *material* artifacts and also for understanding it when in use in the real world (ibid).

**Fig. 2:**
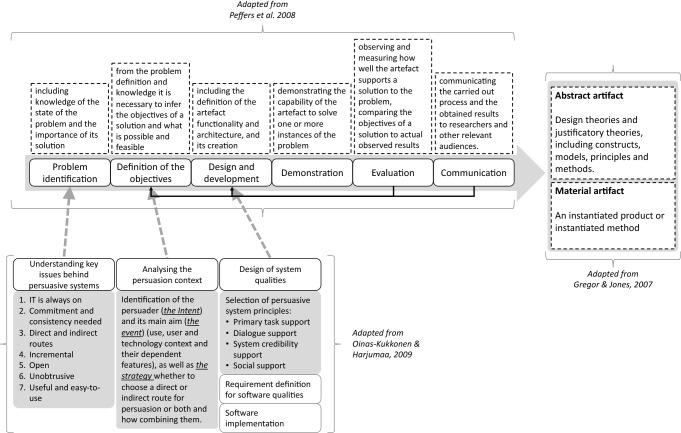
An integrated design science research framework

Peffers et al. (2008) proposed a Design Science Research Methodology (DSRM) as a holistic design science process robustly anchored on prior IS literature (Gregor and Jones 2007; Hevner et al. 2004; Joseph et al. 2004; Walls et al. 1992). As depicted in Fig. 2, this paper adopts a framework based mainly on the Peffers et al. model, in which the contributions of Oinas-Kukkonen and Harjumaa (Oinas-Kukkonen and Harjumaa 2009) and Gregor and Jones (Gregor and Jones 2007) are integrated for taking into account respectively the characteristics of a persuasive system, and the two main outcomes of this design process: the mobile application (the material artifact) and the training model (the abstract artifact).

The six steps identified by Peffers et al. (2008) are used as guideline for describing the proposed integrated framework shown in Fig. 2.

The first step is the *problem identification and motivation*—including knowledge of the state of the problem and the importance of its solution. In this phase, it is important to take into consideration the key elements inherent to persuasive systems (Oinas-Kukkonen and Harjumaa 2009). First, information technology is never neutral, and it is constantly influencing people's behavior. This influence manifests itself in the form of a continuous process rather than a single isolated act. Second, people like to act in an organized and consistent context, and a persuasive system should support such aspects (principles of commitment and cognitive consistency). Third, it is opportune to have a persuasion strategy based on a combination of direct and indirect approaches (blending explicit instructions and subtle hints). Forth, it is easier to persuade people to behave differently through a series of small stimuli rather than through one action (the process of persuasion is often incremental). Fifth, persuasive systems should always be open and make any design bias transparent. Last, it should aim to be not obtrusive (avoid disturbing users while performing their core activities), easy to use and useful to respond properly to the needs of the user.

The second step is the *definition of the objectives for a solution*—from the problem definition and knowledge it is necessary to infer the objectives of a solution and what is possible and feasible. Usually, it is needed to take into account the knowledge of the state of problems and current solutions, as well as the analysis of the persuasion context, in particular the purpose, the event and the strategy (Oinas-Kukkonen and Harjumaa 2009). The purpose or intent regards the persuader identification and the type of change. The persuader is who creates, distributes or adopts technology with the intent of influencing the attitude or behavior of others. The type of change you want to induce may be temporary (easier to obtain) or permanent. The event regards the consideration of (i) the context of use, considering its characteristics; (ii) the context of the user, the individual differences that may affect the interpretation and processing of information, such as individual goals and motivations; (iii) the technological context, the strengths and weaknesses as well as the potential risks and opportunities of the technologies to adopt and their functionalities. The strategy defines the type of message and the type of approach. When defining the message, one must keep in mind the differences between convincing and persuading, in the first case it is based on rational assumptions, in the second case on emotional aspects. The approach, on the other hand, can be direct or indirect (usually adopting a combination of the two approaches), according to the user's characteristics. The aim of this project is to foster an atomic competence acquisition (the *intent*), by defining personalized atomic competence and exploiting several mobile app elements (the *context*) incorporating Fogg’s persuasive system principles (the *strategy*).

The *design and development* represent the third step, in which there is the creation of the artifact. This can be described as a technical or social innovation (Hevner et al. 2004; van Aken 2004), or as “new properties of technical, social, and/or informational resources” (Järvinen 2007). This step includes the definition of the artifact functionality and architecture, and its creation. For including the features of a persuasive system, it is necessary to take into account the design principles that specify the macro-characteristics that such a system must have in order to influence behavior. The design of a persuasive system must be inspired by the principles of support for the main task, user-system dialogue, system credibility and social support. Indeed, the persuasive system must be designed to support the user in carrying out his task, enabling him to simplify, simulate, personalize, reproduce, and monitor learning. The system must be interactive, establishing a dialogue between the user and the system, mainly through verbal or other feedback, to suggest and remember activities, and to recognize and reward the results obtained. The system must be credible for the user to be more convincing, and ultimately motivate the user by leveraging the pressure and influence of the social context (Oinas-Kukkonen and Harjumaa 2009). Specifically, for this project, we design and develop an ad hoc mobile app for supporting the STM for an atomic competence acquisition (*primary task*). The mobile app is able to interact with the user sending different kind of information, such as notifications, messages, reminders, suggestions and rewards (*Dialogue*). The interaction with the mobile app is simple and involve the user for a short period of time (a few minutes), it is quite responsive, it has a reasonable GUI, and uses a secure connection (*System credibility*). Finally, the app includes functionalities for fostering the social engagement such as championships, challenges and open-badge sharing (*Social support*).

The fourth step refers to the *demonstration* of the capability of the artifact to solve one or more instances of the problem (e.g. through experimentation, simulation, case study, proof, or other appropriate activity). This step is followed by the *evaluation* phase that aims to observe and measure how well the artifact supports a solution to the problem, comparing the objectives of a solution to actual observed results. The evaluation could take many forms, quantitative and qualitative (e.g. a comparison of the artifact’s functionality with the solution objectives from step 2, objective performance measures, satisfaction surveys, client feedback, or simulations). At the end it is possible to decide whether to iterate back to step 3 (design and development) attempting to improve the effectiveness of the artifact or to leave further improvement to subsequent projects. Finally, *communication* is the last step, it is suggested to communicate the carried-out process and the obtained results to researchers and other relevant audiences.

According to Gregor and Jones (2007), a design research methodology should produce both an abstract and a material artifact. Therefore, at the end of the six steps recommended by Peffers et al. (2008) and combined with Oinas-Kukkonen and Harjumaa’s work (2009), this research aims at producing both. The abstract artifact has no physical existence and is often described in words or depicted by using images, diagrams, frameworks or other representations. In this design science research project, the abstract artifact is twofold: (i) a specific training method for acquiring an atomic competence and (ii) the process for creating the exercises used for the training, starting by defining the specific atomic competence.

The material artifact, which has a physical existence in the real world, such as a hardware components or a software application (Gregor and Jones 2007), will be instantiated with the mobile application, implementing the logic of the designed training method.

The following section provides a description of the design and development of the mobile app implementing the sequential training method.

## Design and development of the app

**Fig. 3:**
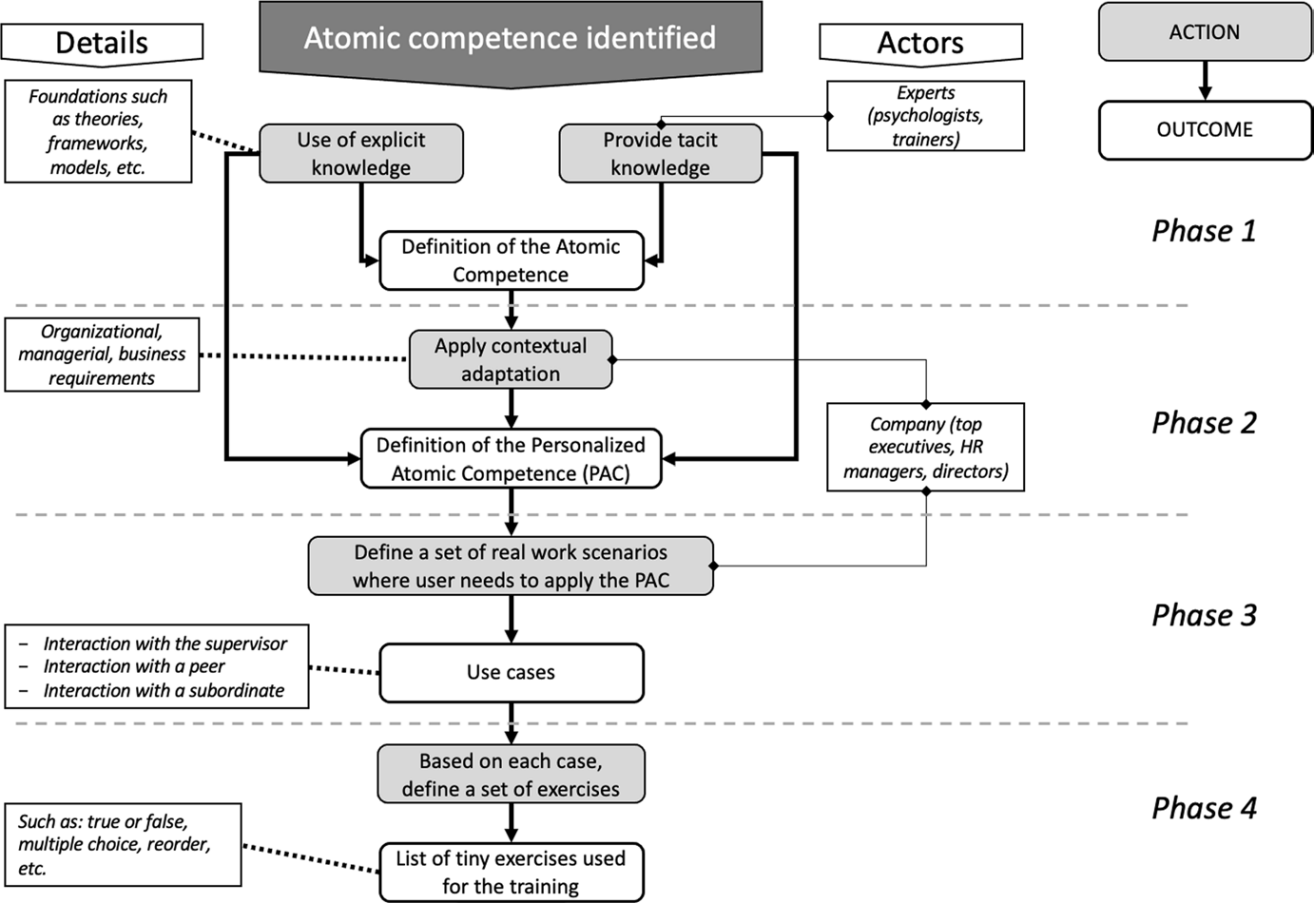
Process for defining the atomic competence and the relative set of exercises

The mobile app has been developed as a persuasive system for supporting individuals to aquire a specific competence from “unconscious incompetent” to “unconscious competent” (Howell 1982). As persuasive system, during this learning process, we ensure that the three factors suggested by Fogg (2009a) were present simultaneously to reach the target behaviour. *Trigger* was incorporated in the mobile app by implementing a notification system. This system could be used to remind the user to perform the exercise on a daily basis as well as to notify if there was an invitation for a challenge or to award achievement. *Motivation* was achieved by exploiting the hedonic and gamification nature of mobile apps (Landers 2015; Shchiglik et al. 2016). To ensure *simplicity*, we decided to develop a series of short exercises relying on the concept of atomic competence. Some examples of the gamification functionalities could be represented by the possibility for a user to invite another one for a challenge (performing the same set of exercises, increasing the time spent on the training), or to obtain and share open-badges related to specific achievements (such as the number of points scored, time spent on the training, number of challenges won, consistency, and much more). Concerning simplicity, it is strongly linked with the definition of the atomic competence that is the target of the training process. Figure 3 describes the protocol adopted for guiding the production of the tiny exercises starting from the identification of the specific atomic competence.

1. Once an atomic competence is identified, we defined it recognizing its main characteristics, using a knowledge base (such as scientific papers and books) and involving training experts. The output of this phase is the definition of an *atomic competence*.
2. In the second phase, we discussed with the organization representatives (e.g. managers or directors) interested in fostering the competence acquisition among their employees. Some customizations on the main characteristics of the atomic competence were introduced where needed to better fit the organizational culture. The output of this phase is the definition of a *personalized atomic competence*, strongly related to the context of application. This definition encompasses the elements that will be used for evaluating its final acquisition by employees.
3. In the third phase, a set of use cases are created in which user should apply that atomic competence in different scenarios, such as when they interact with their direct supervisors, colleagues and/or subordinates. Also, in this case a feedback from the company is relevant.
4. Finally, in the fourth phase, on the basis of the use cases defined previously, a set of tiny exercises were developed, such as true or false questions, multiple choice questions, reordering sequences, etc. These exercises need to be simple, clear, and with an objective understanding (avoiding any form of subjectivity or personal interpretation).

**Fig. 4:**
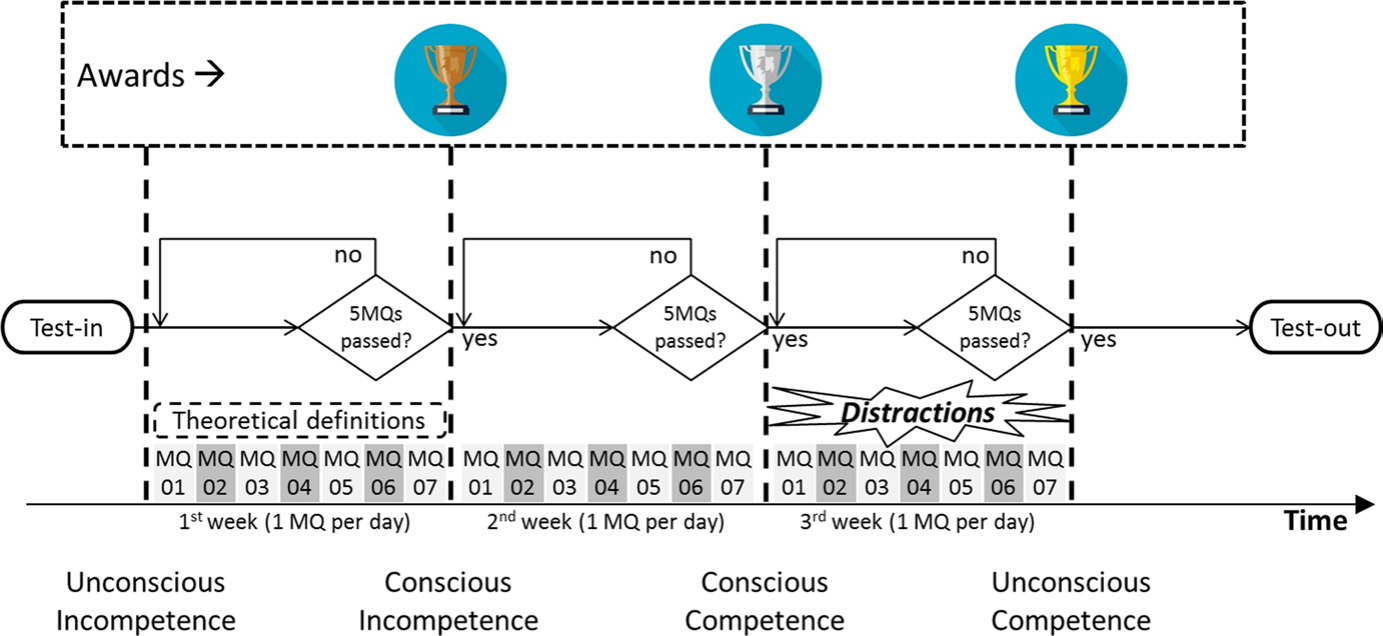
The implementation of the sequential training method (STM)

Once the set of exercises are produced, they are used for training individuals during a pre-defined period of time (e.g. three or four weeks) by performing one small set of exercises every day. There is a correspondence between the number of exercises completed successfully and the level of “consciousness” of the specific competence. Users develop the competence following a step-by-step structure, representing the sequential training method (STM)—starting from initially becoming aware of their current incompetence, then acquiring and applying it consciously, to finally executing this competence autonomously (Fig. 4).

Once the set of tiny exercises is produced for a specific atomic competence, it is necessary defining the three intervals of time (phases) in which the individual (a) becomes aware about his/her incompetence (phase 1), (b) acquires the competence (phase 2) and (c) applies it in an autonomous way (phase 3). According to Fogg (2003, 2009b) the aim is to foster a short daily exercise. It is also suggested that one-week intervals would be an appropriate duration of each phase. In addition, the progress on each phase must be empirically verified during the pilot study of the design process.

Moreover, to attain an unconscious competence level, individuals need to be capable of applying the new skill autonomously. This means that they will perform a desirable behaviour even though there are “distractions” (e.g. the presence of a count down during an exercise, a random flashing alert or physical vibration of the device).

Finally, the app was designed to challenge the user on a daily basis with a set of ten tiny exercises (called “*micro-quest*”). The set of exercises in each micro-quest is randomly selected and there is no duplication during the entire training process. Every time a user completes a micro-quest (MQ) s/he receives a feedback and a score. The micro-quest is considered passed if 80% of the answers are correct. Users can redo the micro-quest until they pass it (exercises are not repeated if a micro-quest is retaken). Users have also the option to not perform a micro-quest. Hence, the micro-quest can have three states: passed, failed or expired (if the user doesn’t perform the daily micro-quest at all). In each STM phase, users need to pass at least five out of the seven micro-quests available during the week. If a user passes less than five micro-quests s/he will be required to restart the specific phase. At the end of each successful phase, users receive an award: bronze, silver and gold medal respectively in the first, second and third phase. The duration of the entire training process is set for three weeks by default (one week per each STM phase). A test is provided at the beginning, as well as at the end of this process in order to measure the effectiveness and efficiency of the training method (Fig. 4).

The app also allows a user to challenge other users on an additional micro-quest. It can also group the people involved in the training process in two or more teams and the score of each team is calculated based on the scores of each member, creating a sort of *championship* as further element of the gamification factor.

**Fig. 5:**
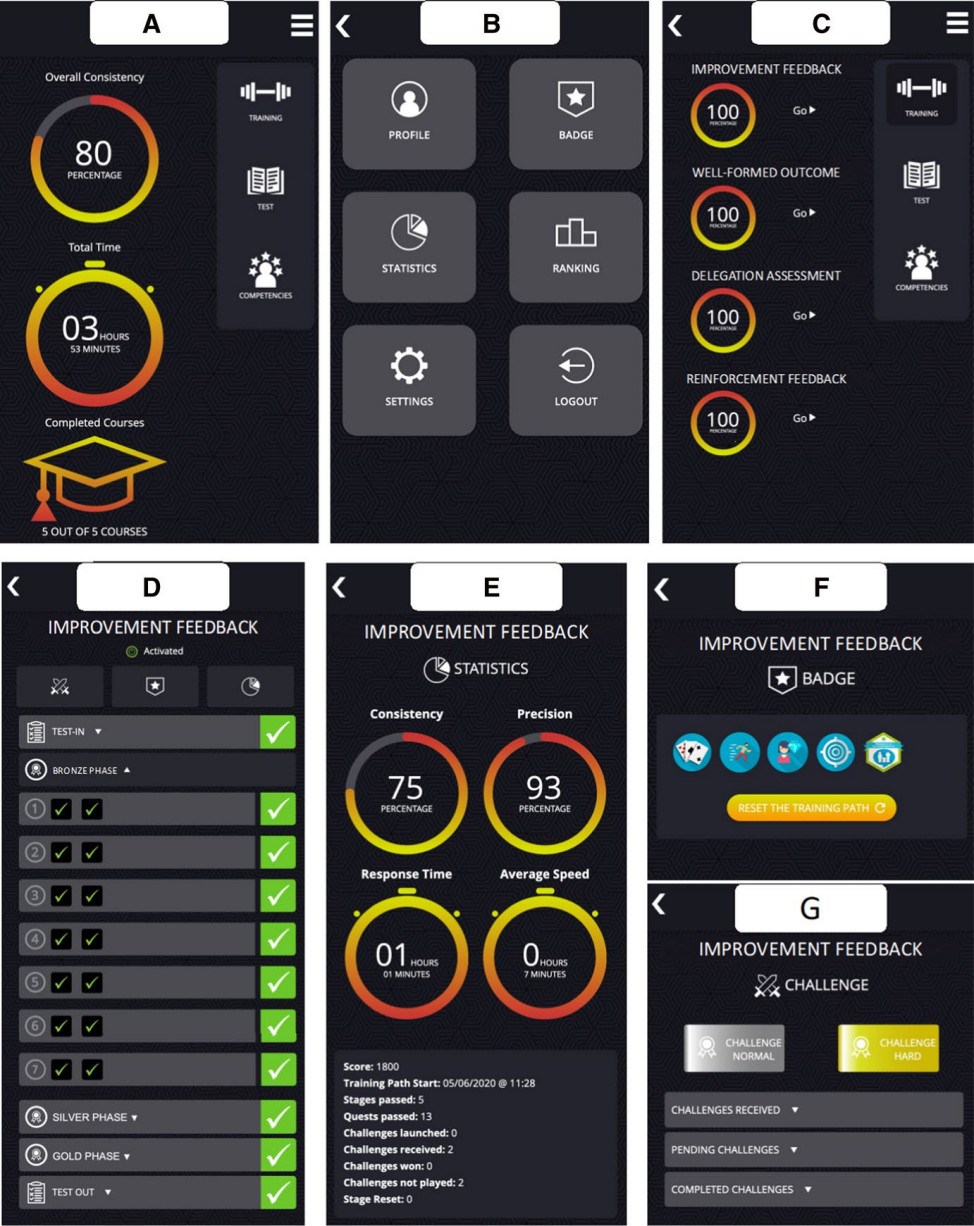
Screenshots of the developed mobile app

Figure 5 shows a set of screenshots highlining some of the persuasive system elements of the mobile app (Oinas-Kukkonen and Harjumaa 2009). Specifically, it shows the main menu (A), the settings section (B), and the overview on training completion (C). These set features enable the users to personalize and monitor their learning activities and outcomes. Figures 5D–G shows some functionalities and features related to the training of the specific atomic competence (improvement feedback concerning): structure of the STM (D), detailed statistics on the learning activities and outcomes (E), and some gamification aspects such as badges (F) and challenges (G).

The next section describes the pilot study conducted in a company to test the efficiency of the STM approach for fostering a specific atomic competence acquisition.

## The pilot study

### Developing the training for an atomic competence

The first step necessary for creating the set of specific exercises for the STM was to identify a specific competence to be acquired by employees. As stated in the introduction, among the top 10 skills that learning and development experts deemed important in 2021, 9 out of 10 were soft skills (Linkedin Learning 2021). Soft skills are “intangible, nontechnical, personality-specific skills that determine one’s strengths as a leader, facilitator, mediator, and negotiator” and are broadly applicable independently of one’s profession (Robles 2012, p. 457). The author points out that the most important soft skills perceived by business executives are integrity and communication. As a result, it was decided to develop a STM focused on the skill of how to communicate an “*improvement feedback*” when an employee or colleague behaves in an undesirable manner.

The result of the first two phases of the framework guiding the production of the tiny exercises (Fig. 2) is the definition of this specific atomic competence, specifically how to communicate an improvement feedback. An “improvement feedback” is not something to be done intuitively, but it represents a skill that needs to be acquired especially by managers who supervise a team of workers. It should be provided in four sequential steps (Aharon Tziner and Latham 1989; Blanchard and Johnson 1983; Pearce and Porter 1986; Shute 2008): (i) the description of undesirable behaviour (that is the reason for providing the “improvement feedback”), (ii) its possible negative consequences (disadvantages), (iii) the description of the desirable behaviour (the alternative), and (iv) its possible positive consequences (advantages). The feedback must be referred to the behaviour and not to the person, and it must be free by the influence of personal opinion. It also should not be based on a comparison with what is done by other people and must be based on objective information or direct observation. Finally, the improvement feedback must be clear and shall not contain contradictions.

These factors were grouped in two specific categories: objectiveness and morphology. Morphology refers to users’ understanding of improvement feedback nature and structure (completeness, order, focus on behaviour and specificity), while objectiveness refers to the impartiality of the manager (judgment based on direct experience and observation, and not based on a comparison with other people, not subjective or does not contain contradictions).

Based on this definition, around 70 use cases were developed. From each use case it was possible to produce 7 or 8 different simple questions. Finally, approximately 500 simple exercises were developed for this specific atomic competence. Most of the exercises provide a description of a simple real situation in which a hypothetical manager needs to give feedback to an employee or a colleague. Few exercises provide theoretical short definitions on the “improvement feedback” used for the first part of the training process (Fig. 3). The exercises were presented in the following formats:

- read and repeat: the text is visualized character by character (as if using a typewriter). Once the entire text is shown on the screen the “OK, I read it! /next” button appears. Usually, this exercise is mostly adopted in the first phase since it provides some definition on the specific atomic competence.
- true or false: provides a question to verify if a feedback is elaborated correctly.
- multiple choice: a feedback example is provided, and the user is asked to identify what specific characteristics or components are missing in the example.
- reorder: this exercise provides the user with a few feedback sentences (components) and asks s/he to organize them in the right order.
- complete: in this exercise an incomplete feedback is provided. The user is asked to select one or more sentences to correctly complete the feedback formulation.

The effectiveness of the mobile app was pre-tested by a group of six people (3 training experts, 2 researchers and one developer). This test group simulated an entire learning process for the specific “soft skill” and thus gave us the possibility to evaluate the functionalities of this method and to refine some aspects about the usability of the app (e.g. default values settings), bugs on the app (user interface) as well as the server side (administration interface).

### Results of the pilot study

**Fig. 6:**
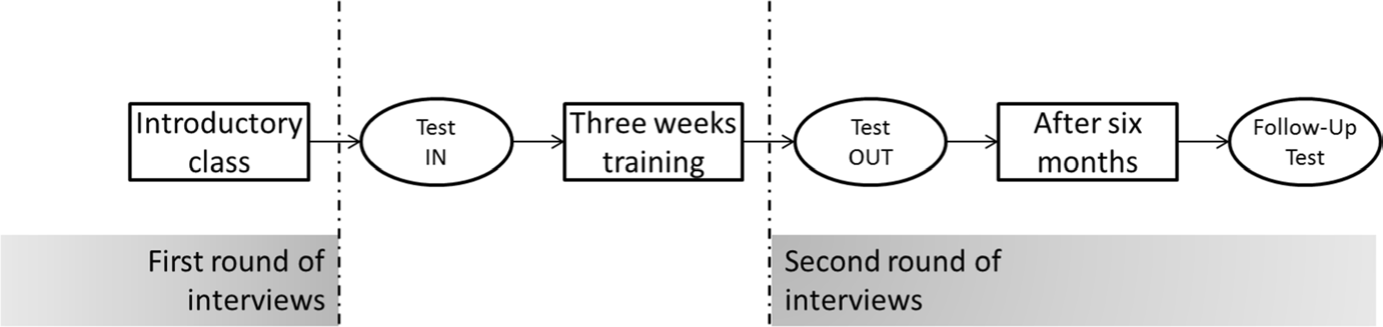
The evaluation process

The study was based on a real-life scenario and was developed to evaluate the overall effectiveness and efficiency of the app. Twenty employees (middle or project managers) of an IT consulting firm were invited to take part in the pilot study. The participants were divided in two groups: a test group consisting of 12 members and a control group with the 8 remaining participants. The participants of the test group were on average 46 years old, while the participants of the control group were 43 years old. The majority of the participants were male, having only one female in each group. While this could potentially represent a gender balance issue of the sample, it is actually a fair representation of gender distribution in the field of software development (https://survey.stackoverflow.co/2022/#developer-profile-demographics). As shown on Fig. 6, both groups started the learning path together in an introductory class, as generally done with blended learning where there is a combination of online and face to face experiences (Means et al. 2013). This class introduced the basic concepts on “how to provide improvement feedback”. During the lesson, several examples were provided based on common scenarios—strongly linking with the work activities that the group was involved in. At the end of the class both groups performed the test for measuring their entrance level (test in). During the following three weeks, both groups received by email multimedia material (e.g. slides or small videos) with messages that reinforced specific concepts covered in class. These messages also encouraged them to apply those concepts in their work environment. In addition to the emails, the test group members had access to the mobile app. On average, half of the app users completed the daily *micro-quest* twice (aiming to improve their scores). In total, more than 85% of the available poll of *“micro-quests” were* utilized during the experiment.

At the end of this follow-up period, all employees involved in the pilot study performed a test (test out) in order to assess if there was any improvement of their competence level. Both test-in and test-out are based on three open-ended cases. Each case provides the description of a hypothetical scenario where an employee produces undesirable behaviour. The user is challenged to provide an improvement feedback for each of the three cases (providing feedback to distinct hierarchical levels—e.g. a peer, a subordinate and a boss).

**Fig. 7:**
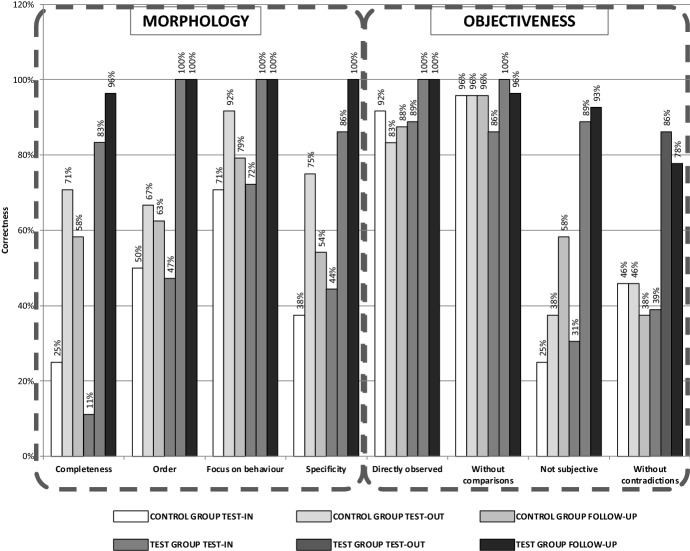
Test-IN, Test-OUT and FOLLOW-UP results comparison between control and test group

The eight factors defined in the training development phase (e.g. structure, nature and objectiveness of the feedback) were used to assess the degree of competence acquisition by the sample. The test-in and test-out results are reported in Fig. 7 for both groups.

The efficacy of the two post-lesson training approaches (with and without the mobile app) was notable by an overall improvement in the scores of the test-in and test-out results. It was also noticeable that the level of improvement on the results of the group using the app was significantly higher. Indeed, the average improvement for the control group was 15.6% while for the test group was 40.6%. This divergence is quite salient regarding the categories measuring feedback objectiveness. In addition, members of the test group achieved a final score of 100% in four categories. On the other hand, the control group did not achieve a score 100% and in one of the criteria (“Directly Observed”) the actual test out score was lower than the test in.

A further follow up test was done six months after the conclusion of the experiment—aiming to evaluate the longitudinal efficacy of the training method. The structure of the assessment was the same used in the test-in and test-out phases, however, different case scenarios were deployed. The results of the follow-up are in line with the outcome of the previous test-out phase, which favoured the test group. In addition, it was noted that some discrepancies between the two groups have increased over time. In particular, the performance gap of the feedback structure parameters had significantly increased between the two groups over time. Overall, the control group presented an average performance decrease of -4.2%, while the test group achieved an average improvement of 2.3%.

Finally, in order to have a more wholesome understanding on the efficacy of this training method, a short feedback was asked to all members of the experiment, aiming to capture their perceptions regarding feedback competence level, efficacy of the training process as well as frequency of use of the competence acquired.

Most of the respondents from the test group noticed positive changes in the feedbacks transmitted by their boss, while most of the control group did not notice any change. Some test group members also observed an improvement in the way the feedback is communicated. One commented: “*I acknowledge positive changes regarding the communication aspects and attention to feedback*.” Another positive result concerns the relationships between feedback providers and feedback receivers. One employee mentioned: “*I saw him (the company owner) more available to answer my questions, especially without creating those situations of embarrassment when the answer to my question could be ‘this is something you should already know’*”. Finally, another respondent noticed a more constructive feedback approach adopted by his managers: “*During the last few weeks, I’ve noticed a more constructive attitude in the way feedback was given to us*”.

As revealed by the participant feedbacks, the perception of the competence level as well as the efficacy of the training method, were increased for all members of both groups—even though it was higher for the test group. The test group feels quite capable of correctly performing the acquired competence, while the control group revealed a certain hesitation on their perceived capabilities to deliver feedback. In addition, it was noted that employees perceived improvement in the feedback given by their managers.

The next section describes the full-scale deployment of the mobile app with a partner organization.

## Case study: supermarket chain

Adopting the same approach described in the pilot study, further training paths concerning other atomic competences were developed. The set of training paths, including the one concerning the improvement feedback, was used for developing full-scale deployment of the mobile app with a partner organization, specifically a supermarket chain. There were 353 users involved, in which 111 were district managers and 242 floor staff (see Table 1).

**Table 1:** Demographic information about District Managers and Floor Staff

Age	District Managers			Floor Staff			Total
	F	M	Sub total	F	M	Sub total	
Under 30	0	0	0	1	0	1	1
30–39	6	7	13	16	21	37	50
40–49	11	9	20	37	23	60	80
50–59	42	24	66	63	52	115	181
Over 60	5	7	12	10	19	29	41
Total	64	47	111	127	115	242	353

**Fig. 8:**
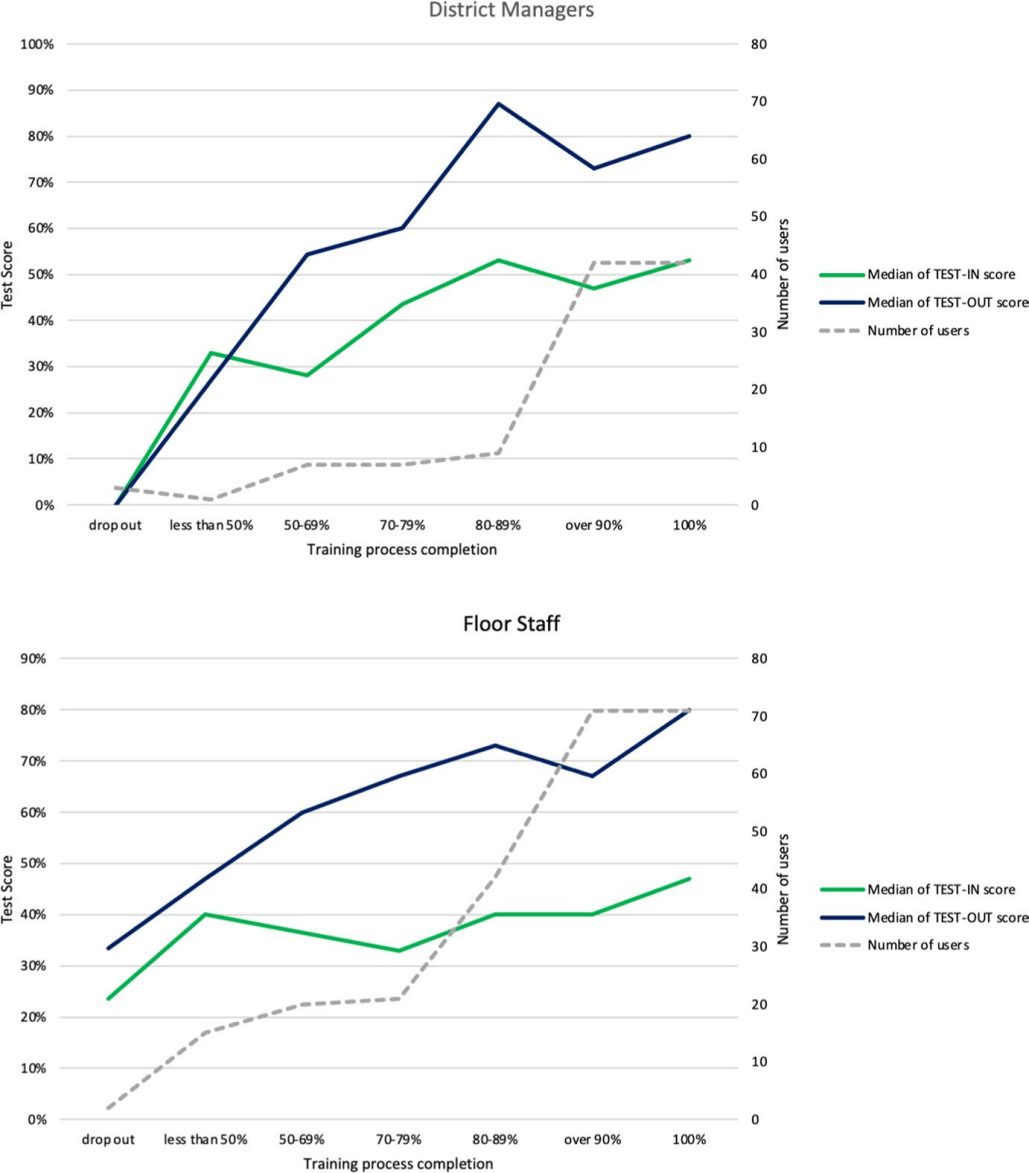
Test-IN and Test-OUT results of District Managers and Floor Staff

All participants were involved in a training process which included the use of the app. For each atomic competence, user performed a Test-IN at the beginning and a Test-OUT at the end of each learning path. An introductory class was performed before the Test-IN and a final class before the Test-OUT. The training process was spread across three weeks’ time, and it was composed by 21 *micro-quests* (MQs). Users had one MQ per day. They were free to choose the frequency they performed MQs (e.g. every day). In order to conform to the structure of the STM, they should have successfully completed at least 5 MQs per week (15 MQs in total) corresponding to approximately 70% of training process completion. At the end of the training, every user performed the Test-OUT regardless of whether they have completed at least 70% of the training process. Figure 8 shows the median of the Test-IN and Test-OUT results in relation to the training process completion for both groups of users. During the training process, the average time spent by every user for performing a MQ is less than 5 min (4 min and 43 s for the floor staff and 4 min and 59 s for the district managers), confirming the limited amount of time per day requested by the STM. Moreover, over 90% of the district managers and 85% of the Floor staff completed at least 70% of the training. These results confirm the efficacy of the persuasive systems elements emended in the mobile app in engaging users in the training process. Finally, the diagrams show an increasing difference between the Test-IN and Test-OUT in relation to the training process completion, especially for those completing at least 70% of the course. This is particularly true for the floor staff group. Those results are in line with what revealed from the pilot study, confirming the efficiency of the STM approach for fostering a specific atomic competence acquisition.

## Discussion and conclusion

This research has taken a multidisciplinary approach by combining research from the Information Systems and Human Resources disciplines. More specifically, in order to develop an integrated framework for designing an IT artifact aimed at fostering competence acquisition, it has its theoretical foundations grounded on Design Science and Employee Development research.

On a theoretical level, this research contributes to Design Science Research in four different ways. First, by contributing to the design knowledge (vom Brocke et al. 2020), developing an integrated DSR framework that combines Peffers et al. (2008) model with the contributions of Oinas-Kukkonen and Harjumaa (2009) and Gregor and Jones (2007). Second, by developing both *abstract* and *material* artifacts. The former is composed by two key elements: (i) a specific training method for fostering competences acquisition (representing mainly the “format”, and together with the gamification elements contributing to the ELM peripheral route) and (ii) the process for creating the exercises used for the training, starting by defining the specific atomic competence (representing the *content*, contributing to the ELM central route). On the other hand, the *material* artifact is the mobile app for facilitating competence acquisition. Thus reaching the dual goals of DSR that are artifact development and knowledge production (Gregor and Jones 2007; Hevner et al. 2004; Peffers et al. 2008; vom Brocke et al. 2020). Third, the mobile app development also relies on and advances the application of two kernel theories (Persuasion Theory & Five Stage Competence Model). The mobile app was not only developed but also tested empirically in a pilot study with workers from an IT consulting firm and a full-scale deployment in a supermarket chain. As such, the framework provides a novel perspective on IS-supported competence acquisition and it can be generalized, to a certain extent, to the development of mobile apps for competence acquisition in the workplace.

Fourth, in order to ensure the robustness of the material artifact and its validity, Fogg’s three fundamental characteristics for persuading individuals to perform a target behaviour (ability, motivation and trigger) (Fogg 2009a) were incorporated in the design through a set of functionalities (notifications, gamification, micro-quest scenario with exercises). The micro-quest especially represents an original approach to foster competence acquisition.

This study also contributes to research on mobile learning and employee development as it focuses on the development of effective digital tools to support competence acquisition. It demonstrated the important role that the mobile app played in the competence acquisition process. The ubiquitous learning environment was able to empower users on their own individual learning process—making it a fertile ground for the development of competence acquisition tools. Furthermore, defining the atomic competence is a crucial element for the implementation of the STM method. Indeed, it is a key success factor to have a well-defined set of dimensions that identify the atomic competence—avoiding potential misinterpretation. These dimensions are the basis for the design of the *micro-quests* required for implementing the STM through the mobile app and they must be measurable in order to have the ability to evaluate each one accordingly. While previous research tends to acknowledge the influential role of IT in HR practices, Stone et al. (Stone et al. 2015) also observe that little research has assessed the effectiveness of these technologies. Therefore, this research represents an original contribution on the effectiveness of mobile app for competence acquisition.

On a managerial level, this study makes contribution to practice by applying the developed mobile app into a workplace setting. The evaluation of the app was carried out through a pilot study that tested the training system in a real-life scenario with managers during a three-week period. By evaluating the effectiveness of the mobile app with employees in situ and in a longitudinal manner, this study demonstrates the potential of mobile learning for HR development. Our study also stresses the importance of behavioural competences for IT professionals. Some IS strategy studies have already indicated that successful strategizing is a social process that relies on communication competences (Marabelli and Galliers 2016; Whittington 2014). In order to have success in the IT sector, practitioners need to acquire a broader managerial or interpersonal skills in addition to their technical ones (Joseph et al. 2010). These skills are identified as “soft” or “behavioural”, and “place emphasis on personal behaviour and managing relationships between people” (Coll and Zegwaard 2006, p. 31).

As a UK report states, employees of the future will have to “be open to and take advantage of new and different approaches to learning, for instance self—directed, bite-sized learning, peer-to-peer learning and technology enabled training opportunities” (UKCES 2017). Failing to do so may reflect on the overall resources to support organizational capabilities and competitive advantages (Basten and Haamann 2018; Za et al. 2014). Therefore, this study offers interesting insights for IT developers as well as for HR managers who have to take decisions in terms of IT investment by highlighting the potential effective role that mobile learning can assume. Future of work should rely on technologies and innovative ways of learning, which should encourage organizations and HR managers to rethink their training policy now. Indeed, according to Kaganer et al. (2013) “mobile learning initiatives should be viewed as long term; expected benefits may not come quickly, and management must guide and support learners through the process of evolving their practices.” (p. 68).

Overall, the results of the empirical tests indicate that the use of the mobile app effectively improved users’ learning process outcomes as well as the ability to deploy the new competence. This finding empirically confirms prior research, especially Hamari et al.'s (2014) literature review of 95 studies that indicates that persuasive systems have a positive effect in 54.7% of the papers, a partially positive effect in 37.9% of the papers and a negative effect in 7.4% of the articles. Since only 10,5% of the articles dealing with persuasive systems have learning / education as target behaviour, this study hopefully will encourage further research in this area.

While this paper makes a positive contribution, there are certainly some limitations. One of them could be related to the specific context (software industry) and profiles (e.g. gender bias) of the samples involved in the pilot study as well as the full-scale deployment. In order to overcome this limitation, future research should involve a wide range of users aiming to reflect the changes in demographics across different industries. Another limitation is that this study focused on a single competence. Future studies could examine different types of atomic competences. From a design perspective, additional data should be collected to measure user’s experience with the learning app (e.g., conducting a survey or semi-structured interviews). The information collected will be useful to refine both the method and also the mobile app. Last, in order to better understand the organizational impact generated by the training method, it could be interesting to evaluate if the use of the app is correlated with change on individual key performance indicators (e.g., productivity, satisfaction, etc.).

Finally, it is hoped that the advances made in this paper will stimulate further reflection about how the gradual emergence of fluid digital ecosystems can leverage the development of new forms of employees’ competence acquisition processes.

## Footnotes

1 https://www.worldbank.org/en/topic/edutech/brief/how-countries-are-using-edtech-to-support-remote-learning-during-the-covid-19-pandemic—retrieved on 03/12/22.
